# Nitidine chloride acts as an apoptosis inducer in human oral cancer cells and a nude mouse xenograft model via inhibition of STAT3

**DOI:** 10.18632/oncotarget.20444

**Published:** 2017-08-24

**Authors:** Lee-Han Kim, Sachita Khadka, Ji-Ae Shin, Ji-Youn Jung, Mi-Heon Ryu, Hyun-Ju Yu, Hae Nim Lee, Boonsil Jang, In-Hyoung Yang, Dong-Hoon Won, Hye-Jeong Kwon, Joseph H. Jeong, Seong Doo Hong, Nam-Pyo Cho, Sung-Dae Cho

**Affiliations:** ^1^ Department of Oral Pathology, School of Dentistry, Institute of Biodegradable Material, Institute of Oral Bioscience, Chonbuk National University, Jeonju, 54896, Republic of Korea; ^2^ Department of Oral Pathology, School of Dentistry and Dental Research Institute, Seoul National University, Seoul 03080, Republic of Korea; ^3^ Department of Companion and Laboratory Animal Science, Kongju National University, Yesan, 32439, Republic of Korea; ^4^ Department of Oral Pathology, School of Dentistry, Yangsan Campus of Pusan National University, Yangsan, 50612, Republic of Korea; ^5^ Department of Developmental Biology and Genomics, College of Veterinary Medicine, Seoul National University and Korea Mouse Phenotyping Center, Seoul, 08826, Republic of Korea

**Keywords:** nitidine chloride, oral cancer, apoptosis, STAT3

## Abstract

Nitidine chloride (NC) is a natural alkaloid compound derived from the plant *Zanthoxylum nitidum* and is known for its therapeutic anticancer potential. In this study, we investigated the effects of NC on growth and signaling pathways in human oral cancer cell lines and a tumor xenograft model. The apoptotic effects and related molecular targets of NC on human oral cancer were investigated using trypan blue exclusion assay, DAPI staining, Live/Dead assay, Western blotting, Immunohistochemistry/Immunofluorescence and a nude mouse tumor xenograft. NC decreased cell viability in both HSC3 and HSC4 cell lines; further analysis demonstrated that cell viability was reduced via apoptosis. STAT3 was hyper-phosphorylated in human oral squamous cell carcinoma (OSCC) compared with normal oral mucosa (NOM) and dephosphorylation of STAT3 by the potent STAT3 inhibitor, cryptotanshinone or NC decreased cell viability and induced apoptosis. NC also suppressed cell viability and induced apoptosis accompanied by dephosphorylating STAT3 in four other oral cancer cell lines. In a tumor xenograft model bearing HSC3 cell tumors, NC suppressed tumor growth and induced apoptosis by regulating STAT3 signaling without liver or kidney toxicity. Our findings suggest that NC is a promising chemotherapeutic candidate against human oral cancer.

## INTRODUCTION

Nitidine chloride (NC) is a natural bioactive alkaloid compound isolated from the root of *Zanthoxylum nitidum*, and is known to have anti-cancer, anti-inflammatory and anti-angiogenic activities against various tumors [1–3]. Recently, NC has attracted great attention as an anticancer drug candidate on the basis of several studies demonstrating that it inhibits cell proliferation and induces apoptosis and cell cycle arrest in several tumor types [4–6]. However, the pro-apoptotic effects of NC in human oral cancer cells and the underlying molecular mechanisms have not been well established.

Signal transducer and activator of transcription (STAT) proteins are a family of seven structurally and functionally related proteins that are well known as latent transcription factors. Among the STAT family members, STAT3 plays an important role in many physiological processes, such as cell proliferation, survival and differentiation [7]. STAT3 is activated by phosphorylation induced by either growth factors or cytokines. Its activation triggers phospho-STAT3 to form a dimer, which subsequently translocates from the cytoplasm to the nucleus where it binds specific DNA sequences and induces transcription of target genes such as c-Myc, cyclin D1, survivin and myeloid cell leukemia-1 (Mcl-1)[8]. Recently, Zhuang et al. reported that STAT3 was overexpressed or constitutively activated in approximately 70% of human tumors compared with normal cells [9]. In particular, persistent activation of STAT3 has been reported in more than 95% of head and neck cancers [10]. Our recent study showed that sorafenib, an oral multi-kinase inhibitor, inactivates the STAT3 signaling pathway to suppress mucoepidermoid carcinoma (one type of oral cancer) *in vitro* and *in vivo* [11]. These findings strongly suggest that the development of drugs that can effectively inactivate STAT3 may serve as one of the most promising strategies for the treatment of oral cancer.

Therefore, the purpose of this study was to investigate the functional role of NC in human oral cancer and the mechanism behind its effects. We demonstrated that STAT3 is constitutively phosphorylated in OSCC compared to NOM, and that NC could act as an apoptotic inducer of human oral cancer *in vitro* and *in vivo*. We also revealed that its apoptotic effect was mainly dependent on inactivation of the STAT3 signaling pathway.

## RESULTS

### NC inhibits the viability of oral cancer cells by triggering apoptotic cell death

**Figure 1:**
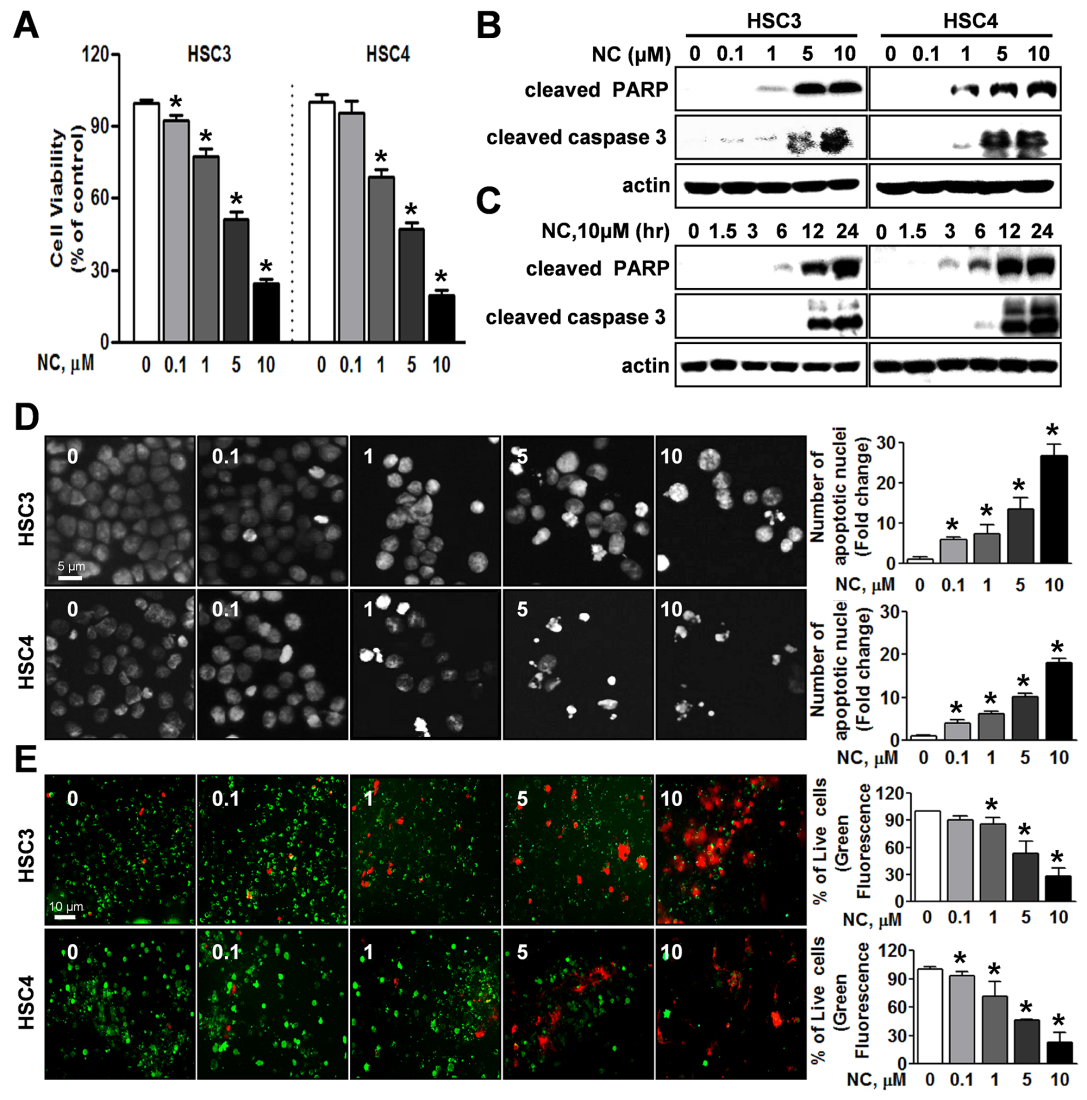
Effect of nitidine chloride (NC) on cell viability and apoptosis in human oral cancer cell lines HSC3 and HSC4 cells were treated with DMSO or various concentrations of NC (0.1, 1, 5 and 10 μM) for 24 hr or with 10 μM of NC for 1.5, 3, 6, 12 and 24 hr. **(A)** The effect of NC on cell viability was determined using a trypan blue exclusion assay. The graphs are expressed the mean ± S.D. of triplicate experiments and significance (*p* < 0.05) compared with the DMSO-treated group was indicated (*). **(B** and **C)** The apoptotic effect of NC was determined by Western blotting with the indicated antibodies (cleaved PARP and caspase 3). Actin was used as an internal control. **(D)** Fluorescence microscopy images of 4’-6-diamidino-2-phenylindole (DAPI)-stained HSC3 and HSC4 cells (magnification, X400). The number of cells with nuclear condensation and fragmentation was quantified. The graphs represent the mean ± S.D. of triplicate experiments. *, *p* < 0.05 is compared with control group. **(E)** Qualitative assessments of NC-induced cell death by a live/dead assay, which differentially labels live (green) and dead (red) cells with fluorescent dyes (magnification, X200). The graphs represent the mean ± S.D. of triplicate experiments. *, *p* < 0.05 is compared with control group.

To determine the potential effect of NC on the viability of human oral cancer cells, HSC3 and HSC4 cells were treated with NC for 24 hr at concentrations ranging from 0.1 to 10 μM. Cell viability was reduced by NC in a concentration-dependent manner (Figure 1A). Both cell lines showed maximum growth inhibition of approximately 20∼30% at a high concentration of 10 μM NC. To decipher the apoptotic effect of NC in these oral cancer cell lines, we performed Western blotting to evaluate the expression of the apoptosis-associated protein [cleaved poly ADP ribose polymerase (PARP) and caspase 3]. Treatment with NC clearly induced the cleavage of PARP and caspase-3 in a concentration- and time-dependent manner (Figures 1B and 1C). Apoptotic morphologic changes after treatment with NC could be detected using 4’,6-Diamidino-2-Phenylindole (DAPI) staining. As illustrated in Figure 1D, both cell lines treated with vehicle control were typically round-shaped and homogeneously stained, whereas cells treated with NC showed nuclear condensation and fragmentation. Moreover, NC treatment generated significantly more red fluorescence in a live/dead assay, indicating that NC induced cell death in HSC3 and HSC4 cells (Figure 1E).These results suggest that NC induces cell death via apoptosis in human oral cancer cell lines.

### STAT3 is hyper-phosphorylated in OSCC and NC triggers apoptosis by inhibiting phosphorylation of STAT3

**Figure 2:**
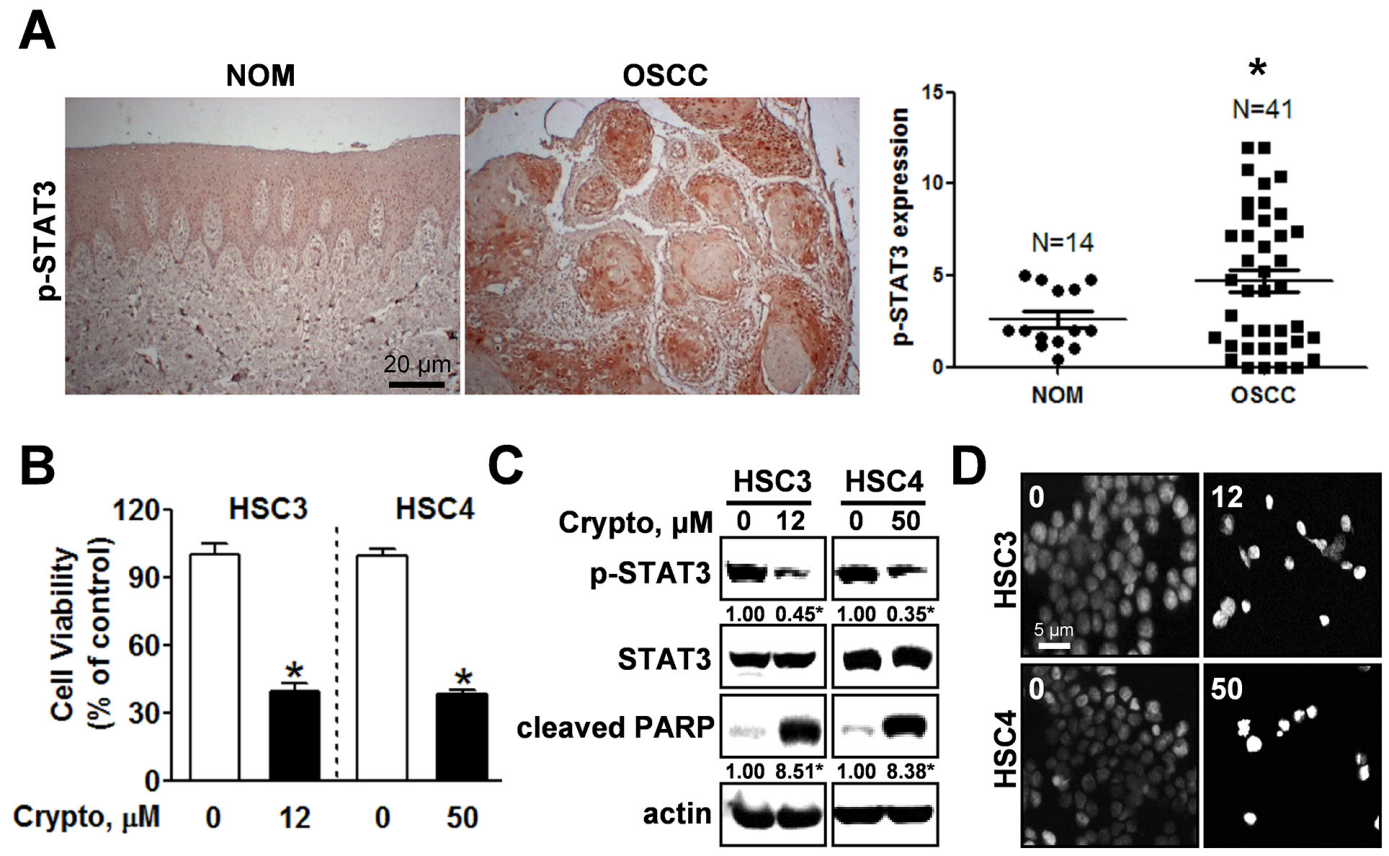
STAT3 is hyper-phosphorylated in OSCC and inhibiting phosphorylation of STAT3 by cryptotanshione triggers apoptosis in oral cancer cell lines **(A)** Left panel: Expression of phosphorylated STAT3 (p-STAT3) was evaluated by immunohistochemistry in tissue samples of patients with OSCC (n=41) compared with normal oral mucosa (NOM, n=14); Right panel: Dot-plot graph of p-STAT3 expression. (*) indicates *p* < 0.05 significant difference between NOM and OSCC group. **(B)** HSC3 and HSC4 cells were treated with DMSO or the STAT3 inhibitor, cryptotanshinone (Crypto, 12 and 50 μM, respectively) and cell viability was analyzed using a trypan blue exclusion assay. The graphs represent the mean ± S.D. of triplicate experiments. *, *p* < 0.05 is compared with control group. **(C)** Whole-cell lysates were analyzed by Western blotting using antibodies against p-STAT3, STAT3, and cleaved PARP. Data represent the mean of triplicate experiments. *, *p* < 0.05 is compared with control group. **(D)** Nuclear condensation and fragmentation were assessed by staining with DAPI (magnification X400).

**Figure 3:**
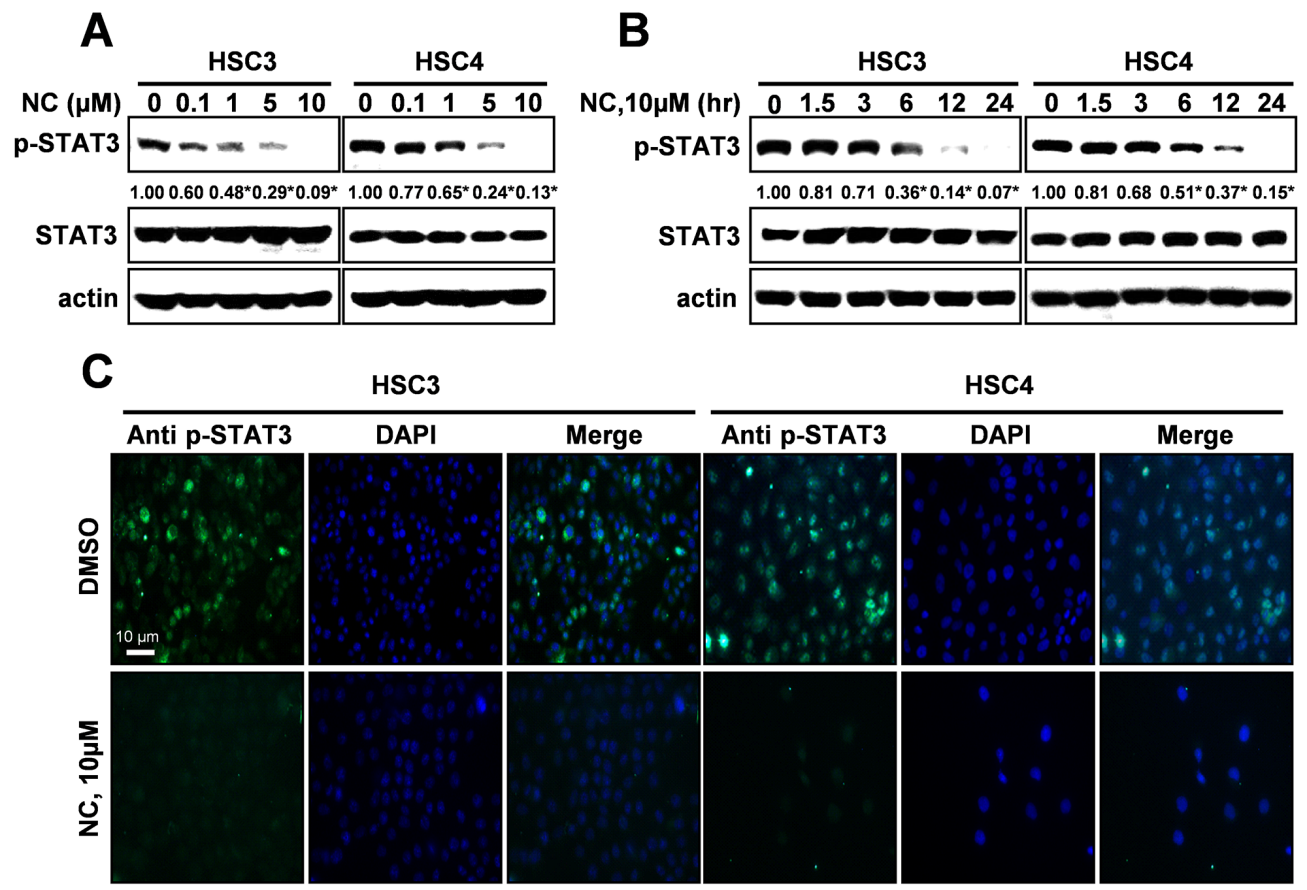
Nitidine chloride (NC) reduces the expression levels of p-STAT3 in oral cancer cell lines The expression of p-STAT3 was determined by Western blotting in a concentration- **(A)** and time-dependent manner **(B)**. Data represent the mean of triplicate experiments. *, *p* < 0.05 is compared with control group. **(C)** p-STAT3 protein expression was confirmed by immunofluorescence using florescence microscopy (magnification X200).

**Figure 4:**
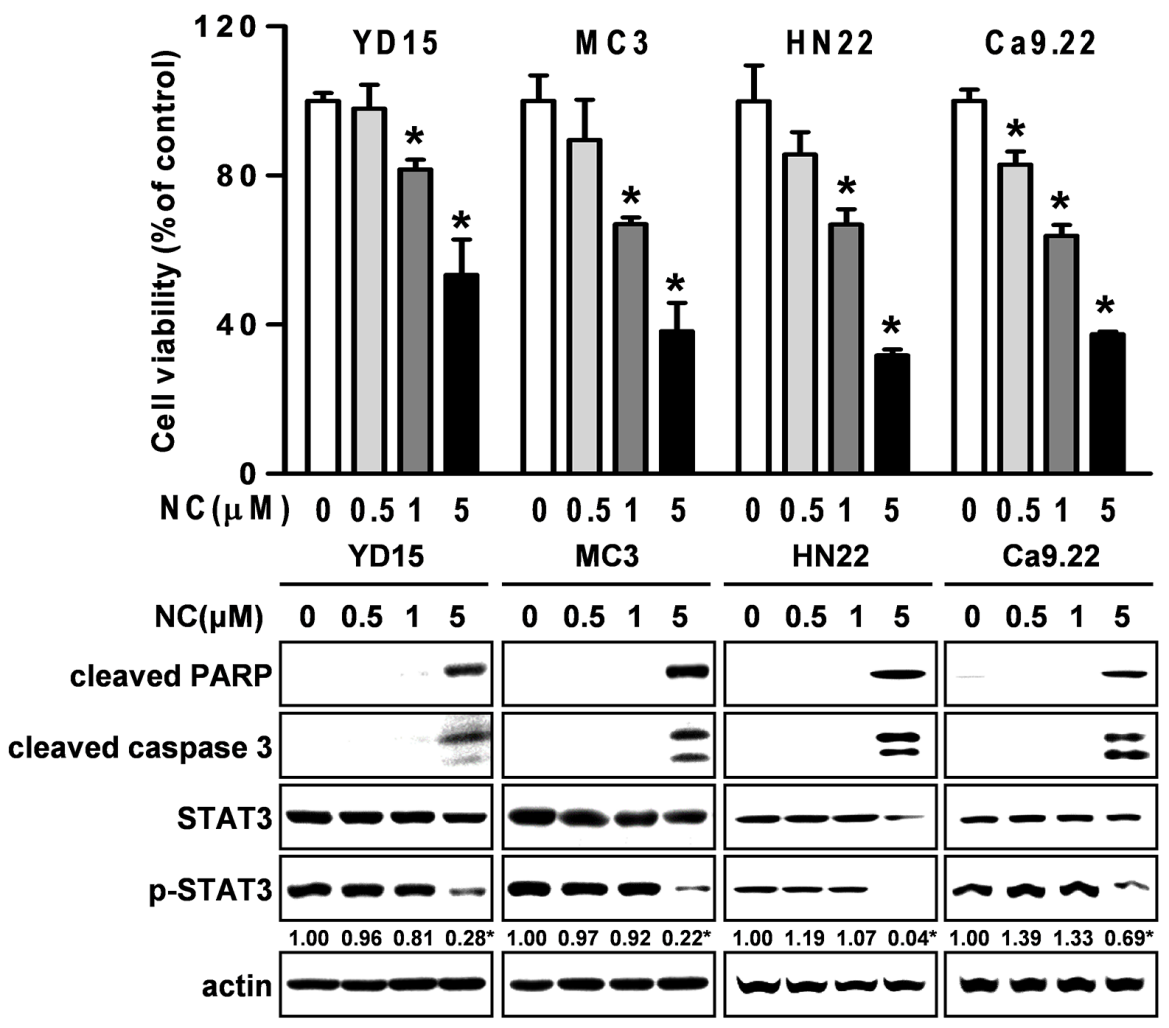
The effect of NC on cell growth and apoptosis in four other different oral cancer cell lines (YD15, MC3, HN22 and Ca9.22) Cells were treated with various concentrations of NC for 24hr. The growth inhibitory effect of NC was examined by trypan blue exclusion assay, and the effects of NC on PARP and caspase 3 cleavages, STAT3, and p-STAT3 were analyzed by Western blotting. The graphs represent the mean ± S.D. of triplicate experiments. *, *p* < 0.05 are compared with control group.

Expression of active STAT3 is known to play an important role in tumorigenesis [12]. We analyzed the phosphorylation of STAT3 in normal oral mucosa (NOM ; n=14) and tissues from patients with OSCC (n=41) and found that expression of phosphorylated STAT3 was significantly higher in OSCC than NOM (Figure 2A). To verify the functional role of STAT3 in oral cancer cell lines, we used cryptotanshinone (Crypto), a potent STAT3 inhibitor [13]. The results in Figures 2B-2D show that Crypto decreased cell viability and induced apoptosis by dephosphorylating STAT3 in human oral cancer cell lines, suggesting that phosphorylation of STAT3 is closely related to oral cancer and might be a good chemotherapeutic target. We further investigated the effect of NC on STAT3 signaling in HSC3 and HSC4 cells. Figures 3A and 3B show that treatment with NC significantly down-regulated the expression of phospho-STAT3 in a concentration- and time-dependent manner. Immunofluorescence staining confirmed the dephosphorylating activity of NC in STAT3 signaling (Figure 3C). In addition, NC suppressed cell viability and increased cleaved PARP and caspase 3 in four other oral cancer cell lines including YD15, MC3, HN22 and Ca9.22 cells by reducing phosphorylation of STAT3 (Figure 4). These results suggest that the pro-apoptotic activity of NC is in part due to inactivation of STAT3. Next, the effects of NC on apoptosis were compared with those of other STAT3 inhibitors such as cryptotanshione and S3I-201 on apoptosis in both cell lines. As shown in Supplementary Figure 1, NC treatment strongly induced the cleavage of PARP suggesting NC might be a more potent apoptosis inducer than other STAT3 inhibitors.

### NC inhibits tumor growth in a xenograft model bearing HSC3 cells

**Figure 5:**
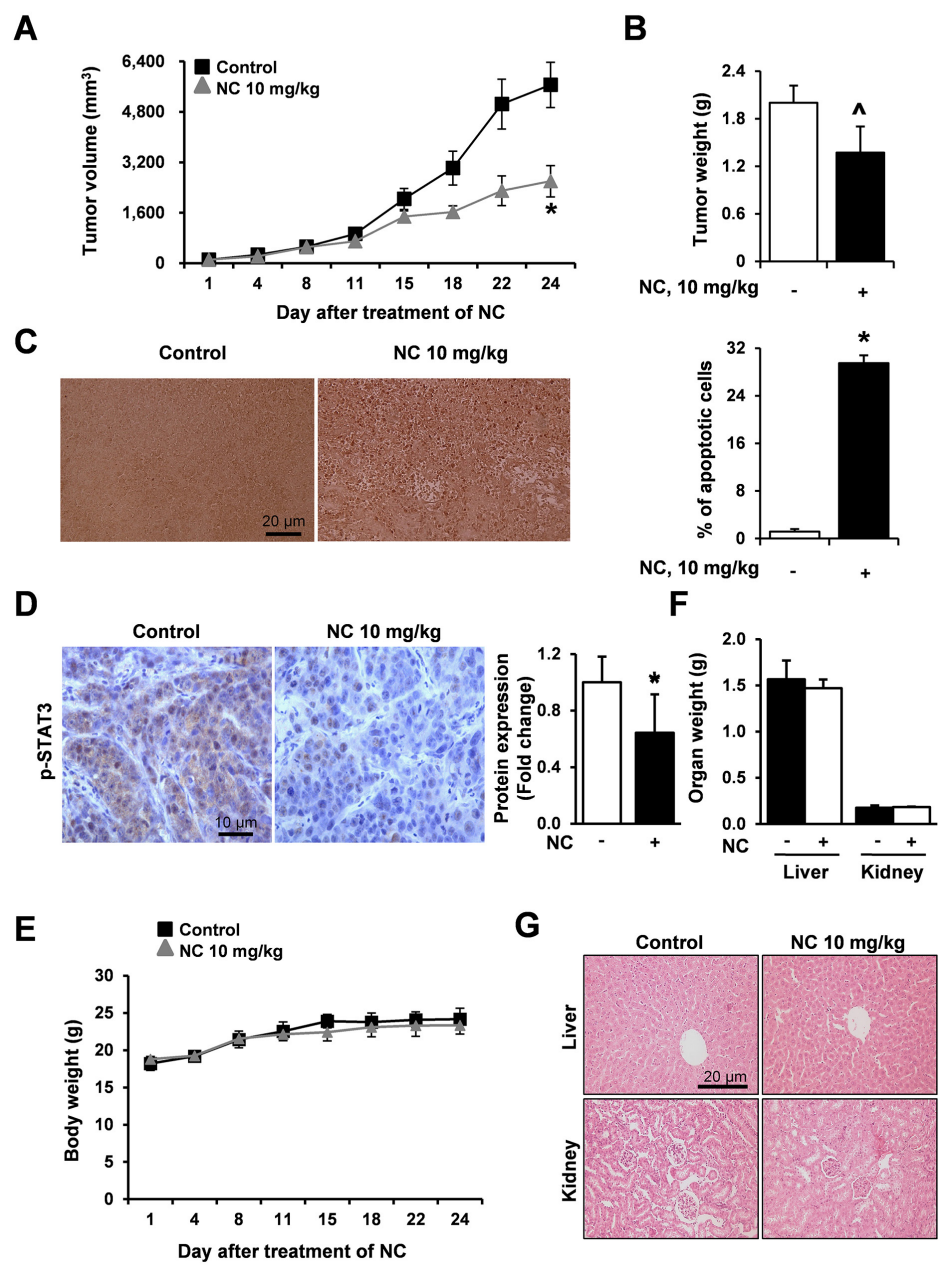
NC suppresses tumor growth and induces apoptosis in a nude mouse xenograft model bearing HSC3 cells Athymic nude mice bearing HSC3 cells (4 mice per group) were treated with vehicle control or NC (10 mg/kg) for 24 days. Tumor volume **(A),** tumor weight **(B)** and body weight **(E)** were calculated at the indicated time points after treatment with NC. The graphs represent the mean ± S.E. of triplicate experiments. *, *p* < 0.05 is compared with control group. ^*p*=0.057 **(C)** Apoptosis was detected in tumor tissues using TUNEL assay (magnification, X200). Results are expressed as mean ± S.E and significance (*p* < 0.05) compared with the control-treated controls are indicated (*). **(D)** The expression levels of p-STAT3 were evaluated using immunohistochemistry. Slides were observed using a microscope (magnification X400). The graphs represent the mean ± S.E. of triplicate experiments. *, *p* < 0.05 is compared with control group. **(F)** Organ weights (liver and kidney) were measured. **(G)** Two organ tissue samples from control- and NC-treated mice were subjected to histopathological analysis using H&E staining (magnification, X200).

To determine whether NC inhibits tumor growth *in vivo*, we injected HSC3 cells subcutaneously into the flank of athymic nude mice. Tumor growth was significantly inhibited in mice treated with NC (Figure 5A) at day 24 and tumor weight was at the margin of significance (p=0.057) (Figure 5B). Results of a terminal deoxynucleotidyl transferase dUTP nick end labeling (TUNEL) assay showed that NC increased the number of TUNEL-positive cells in NC-treated tumors confirming that NC could induce apoptosis of HSC3 cells *in vivo* (Figure 5C). In addition, immunohistochemistry of NC-treated HSC3 tumor xenografts showed that the expression levels of phosphorylated STAT3 were significantly reduced in NC-treated tumors at the protein level (Figure 5D). Our results consistently demonstrated that NC induced apoptotic cell death through inhibition of STAT3 expression *in vitro* and *in vivo*. Moreover, body weight, organ weight and histopathological findings indicated that there was no hepatic or nephrotic toxicity at the dose of NC used in this study (Figures 5E, 5F and 5G).

## DISCUSSION

Numerous natural products have been tested in efforts to discover novel anti-cancer therapeutic agents for the treatment of various tumors because natural agents typically exhibit fewer side effects than currently used anti-cancer chemotherapeutic drugs [14, 15]. Nitidine chloride (NC), a natural benzophenanthridine alkaloid, is a major active compound present in a well-known traditional Chinese medicinal herb *Zanthoxylum nitidum* [16]. Recently, NC has been reported to inhibit proliferation, migration and invasion and to induce apoptosis in several tumors including colorectal and breast cancer [17, 18]. In this study, we demonstrated for the first time that NC exhibits anti-cancer activity in human oral cancers both *in vitro* and *in vivo*, supporting the potential use of NC as an anti-cancer drug for the treatment of oral cancer. To investigate the anticancer activities of NC in human oral cancer cell lines, we examined cell viability and apoptosis by trypan blue exclusion assay, DAPI staining, Western blotting and live/dead assay. As shown in Figure 1, cell viability was obviously reduced by NC in a concentration-dependent manner accompanied by the induction of apoptotic cell death. These data showed that NC at concentrations of 5-10 μM significantly affected oral cancer cell lines. Consistent with our findings, several previous studies also demonstrated that NC at concentration ranges of 5-50 μM clearly reduced cell viability in human renal, gastric and hepatic cancers [3, 16, 19]. We further demonstrated the antitumor activity of NC on oral cancer *in vivo*. NC (10 mg/kg/day for 24 days) significantly suppressed tumor volumes and weights in a nude mouse xenograft model bearing HSC3 cells accompanied by an increase in the number of TUNEL-positive cells (Figures 5A, 5B and 5C), consistent with findings of our *in vitro* study. These data indicate that the antitumor activity of NC on oral cancer is achieved mainly through its apoptotic potential.

Recently, numerous reports have shown that aberrant and persistent activation of the STAT3 signaling pathway contributes to tumor progression [20–24]. Several studies have indicated constitutively activated STAT3 in human tumor samples [25]. In the present study, we clearly demonstrated that STAT3 was significantly activated in OSCC compared to NOM implying that activation of STAT3 plays a critical role in the development of oral cancer (Figure 2A). A number of compounds that inhibit the activity or function of STAT3 have been developed for use in cancer treatment and prevention [26, 27]. For example, 6-bromoindirubin-3’-oxime inhibits STAT3 signaling and induces apoptosis of human melanoma cells [12], and the STAT3 inhibitor WP1066 attenuates miRNA-21 to suppress human oral cancer growth *in vitro* and *in vivo* [28]. Thus, STAT3 might be a potential therapeutic target for the treatment of oral cancer as well as other tumors. Our results showed that either NC or a STAT3 inhibitor inactivated the STAT3 signaling pathway to suppress cell viability and induce apoptosis in human oral cancer cell lines. We also performed the effects of NC on cell viability, apoptosis and STAT3 phosphorylation in four different oral cancer cell lines. The results showed that NC significantly suppressed cell viability and increased the cleavages of caspase 3 and PARP. We also found that NC acts on STAT3 activation. Thus, we strongly suggest that the inhibition of STAT3 is a common effect of NC in human oral cancer cell lines. These data are supported by recent studies demonstrating that NC inhibits cell growth and angiogenesis in human gastric and hepatic cancers through suppression of the STAT3 signaling pathway [3, 16]. Recent reports have demonstrated that NC inhibits tumor cell growth and cell metastasis via c-SRC/Fak, ERK, or Akt-associated pathways in several cancer cells; therefore, we cannot exclude the possibility of involvement of other kinases in NC-mediated apoptosis of oral cancer cells [1, 18, 19].

To evaluate the anti-tumor activity of NC *in vivo*, an athymic nude mouse xenograft model bearing HSC3 cell was used and administrated with NC (10 mg/kg/day). We observed that NC decreased tumor volume and weight, increased the number of TUNEL-positive cells (Figures 5A, 5B and 5C), and showed distinctly reduced protein expression levels of phospho-STAT3 in the tumors of NC-treated mice compared to the tumors of vehicle-treated mice (Figure 5D), consistent with our *in vitro* data. Previously, other groups also reported that NC (5 to 10 mg/kg/day) suppressed tumor growth *in vivo* by repressing multiple signaling pathways in human gastric, hepatic, and renal cancers [3, 16, 19]. These data strongly suggest that NC can suppress tumor growth via the STAT3 signaling pathway both *in vitro* and *in vivo*. Moreover, our observations that NC did not affect body or organ weight and the absence of histopathologic abnormalities (Figures 5E, 5F and 5G) suggesting that NC might not have serious side effects, indicating that there is no major obstacle for further application of NC in clinical trials. To the best of our knowledge, our *in vivo* results are the first demonstration of strong apoptotic activity of NC in human oral cancer with no side effects.

In conclusion, in the present study, we demonstrated that NC significantly inhibited tumor growth and induced apoptosis in human oral cancer cell lines and HSC3 tumor xenografts in mice. In addition, our results revealed that NC inhibited STAT3 phosphorylation in human oral cancer cell lines. These results suggest that NC could be a promising therapeutic agent for the treatment of human oral cancer.

## MATERIALS AND METHODS

### Cell culture and chemical treatment

HSC3, HSC4 and Ca9.22 cell lines were kindly provided by Hokkaido University (Hokkaido, Japan). YD15 and MC3 cell lines were obtained from Yonsei University (Seoul, Korea) and Fourth Military Medical University (Xi’an, China), respectively. HN22 cells were obtained from Dankook University (Cheonan, Korea). Cells were cultured in either DMEM or RPMI1640 media supplemented with 10% fetal bovine serum (FBS) and antibiotics at 37°C in 5% CO_2_ incubator. All experiments were performed with cells cultured at 50∼60% confluence. Cryptotanshinone and nitidine chloride (NC) were purchased from Sigma-Aldrich Chemical Co. (St. Louis, MO, USA). They were dissolved in dimethyl sulfoxide (DMSO), aliquoted, and stored at -20 °C. The final concentration of DMSO did not exceed 0.1%.

### Measurement of cell viability

The effect of NC on cell viability was determined using a trypan blue exclusion assay. Cells were stained with 0.4% trypan blue (GIBCO, Paisley, UK), and viable cells were counted using a hemocytometer. All experiments were performed independently three times with triplicate samples in each experiment.

### Western blot analysis

Whole-cell lysates were prepared with lysis buffer and the protein concentration in each sample was measured using a *DC* Protein Assay Kit (BIO-RAD Laboratories). After normalization, equal amounts of protein were separated by sodium dodecyl sulfate polyacrylamide gel electrophoresis (SDS-PAGE) and transferred to Immuno-Blot PVDF membranes. The membranes were blocked with 5% skim milk in tris-buffered saline with Tween 20 (TBST) at room temperature (RT) for 2 hr and then incubated with primary antibodies and corresponding horseradish peroxidase (HRP)-conjugated secondary antibodies. Antibodies against cleaved PARP, cleaved caspase-3, STAT3, and p-STAT3 were purchased from Cell Signaling Technology, Inc. (Charlottesville, VA, USA). Anti-actin antibody was obtained from Santa Cruz Biotechnology, Inc. (Santa Cruz, CA, USA). The immunoreactive bands were visualized using ImageQuant LAS 500 (GE Healthcare Life Sciences).

### 4'-6-Diamidino-2-phenylindole (DAPI) staining

To detect nuclear morphological changes of apoptotic cells, cells were stained with DAPI solution (Sigma-Aldrich, Louis, MO, USA). Briefly, cells were fixed in 100% methanol at RT for 10 min, deposited on slides, and stained with DAPI solution. Morphologic changes were observed by a fluorescence microscopy (ZEISS Axio Imager, Germany).

### Live/dead assay

The LIVE/DEAD Viability/Cytotoxicity Kit (Invitrogen) was used to determine cell viability. The polyanionic dye Calcein-AM is retained within live cells, producing an intense green fluorescence through intracellular esterase activity. Ethidium homodimer-1 enters cells with damaged membranes and binds to nucleic acids, producing a bright red fluorescence in dead cells. Briefly, cells were stained with 2 μM Calcein-AM and 4 μM ethidium homodimer-1, and then incubated for 30 min at RT before analysis by fluorescence microscopy.

### Patients and tissue samples

Fourteen NOM samples were obtained during third molar removal from adult patients with no pathologic lesions at Pusan National University Dental Hospital (Pusan, Korea). Forty one samples of OSCC tissue with good preservation of paraffin tissue and hematoxylin & eosin–stained slides were obtained from the files of the Department of Pathology, Medical College, Pusan National University between January 1993 and December 2007. Experiments were performed according to the ethics committee approved by Pusan National University, School of Dentistry (PNUDH-2015-014), and written informed consent was obtained from all human subjects.

### Immunohistochemistry (IHC)

Unstained tissue sections were deparaffinized, treated with 100% alcohol, and washed with phosphate-buffered saline (PBS). For antigen retrieval, sections were boiled in citric buffer (pH 6.0) for 10 min using a hot plate and then cooled for 1 hr at RT. Endogenous peroxidase was blocked with peroxidase blocking solution (Sigma-Aldrich) for 10 min, and the sections were treated with protein blocking solution for 20 min. Antibody against p-STAT3 was applied overnight at 4°C, followed by incubation with secondary antibodies for 1 hr at 37°C. Sections were then stained with freshly prepared DAB substrate (Dako), counterstained with Mayer’s hematoxylin, dehydrated, mounted, and examined under a light microscope. To analyze sections, five non-overlapping fields per slide were randomly selected and images were captured with a light microscope attached to a digital camera (Olympus, BX51T, Tokyo, Japan, X100). The captured images were examined independently in a blinded manner by two experienced oral pathologists.

### Immunofluorescence

Cells were seeded on 4-well culture plates and treated with DMSO or NC (10 μM). After 24 hr, cells were fixed and permeabilized using cytofix/cytoperm solution (BD Bioscience) for 1 hr at 4°C. Cells were then blocked with 1% bovine serum albumin in PBS for 1 hr at RT and incubated overnight at 4°C with p-STAT3 antibody. Subsequently, the cells were exposed to the FITC-conjugated secondary antibodies for 1 hr at RT and visualized using a fluorescence microscope equipped with the appropriate filters for DAPI and FITC dyes.

### Nude mouse xenograft assay

Female nude mice were purchased from Orient Ltd (Suwon, Korea). All mice were handled according to the Institutional Animal Care and Use Committee (IACUC) guidelines approved by Kongju National University (Republic of Korea). *In vivo* protocol was modified based on previous studies [3, 6, 16, 19]. HSC3 cells were inoculated by subcutaneous (s.c.) injection into the flanks of the mice. The mice were then randomly assigned to one of two treatment groups (n=4 for each group): the treatment group was received 10 mg/kg/day of NC (intraperitoneal: i.p.) five times per week for 24 days, and the control group received an equal volume of the vehicle (0.1% DMSO). Tumor volume and body weight were measured twice a week. After 24 days, tumor weight and organ weight were measured. The tumor volumes were measured along the two diameter axes with calibers to allow calculation of tumor volume using the following formula: V=π/6[(D+d)/2]^3^, where D and d were the larger and smaller diameters, respectively.

### Terminal deoxynucleotidyl transferase dUTP nick end labeling (TUNEL) assay

Paraffin-embedded tumor tissues were analyzed using a TUNEL *in situ* apoptosis detection kit (Dead-End Colorimetric TUNEL system, Promega) according to the manufacturer’s instructions. Briefly, paraffin-embedded sections were deparaffinized and rehydrated. The sections were incubated with proteinase K for 15 min at RT, the endogenous peroxidase was blocked with 0.3% hydrogen peroxide for 5 min. The digoxigenine-dUTP end labeled DNA was detected using an anti-digoxigenin peroxidase antibody followed by peroxidase detection with 0.05% DAB containing 0.02% hydrogen peroxide. The sections were counterstained with methyl green, and the brown-colored apoptotic bodies in the tumor sections from control and NC-treated mice were counted using a Nikon Eclipse E800 microscope (Nikon Inc.).

### Histopathological examination of organs

Mice organs (liver and kidney) were fixed in 10% neutral buffered formalin. Tissue sections were cut at a thickness of 4 μm and stained with hematoxylin and eosin (H&E). Histopathological changes were analyzed using a Nikon Eclipse E800 microscope.

### Statistical analysis

For two experimental comparisons, a two-tailed *Student’s t-test* was used and for multiple comparisons, one-way ANOVAs were applied. Non-parametric statistics, Mann-Whitney test was performed for the analysis of non-normally distributed data. Statistical significance is represented by asterisks corresponding to * *p*<0.05.

## SUPPLEMENTARY MATERIALS FIGURE


