# Supplementary material for: Nitidine chloride acts as an apoptosis inducer in human oral cancer cells and a nude mouse xenograft model via inhibition of STAT3
Source: Oncotarget. 2017 Aug 24;8(53):91306–15. doi: 10.18632/oncotarget.20444 (PMC5710925; doi:10.18632/oncotarget.20444)
Supplement: Supplementary file 1 [file oncotarget-08-91306-s001.pdf]

Nitidine chloride acts as an apoptosis inducer in human oral cancer cells and a nude mouse xenograft model via inhibition of STAT3

SUPPLEMENTARY MATERIALS

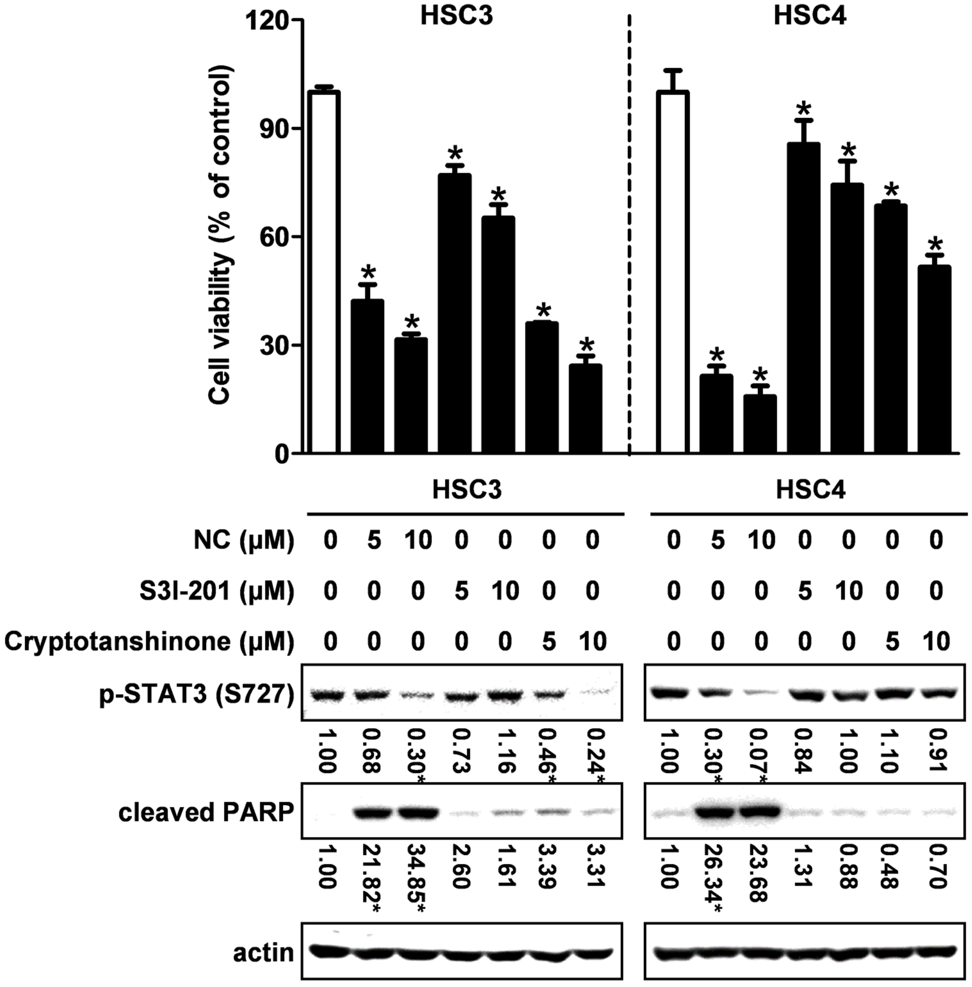

Supplementary Figure 1: The effect of STAT3 inhibitors on cell growth and apoptosis In HSC and HSC4 cells. Cells were trated with same concentration of S3l-201, cryptotanshinone and NC for 24hr. The growth inhibitory effect of STAT3 inhibitors was examined by trypan blue exclusion assay, and the effects of STAT3 inhibitors on PARP cleavage were analyzed Western blot. The graphs represent the mean ± S.D. of triplicate experiments. *, $p < 0.05$ is copared with control group.
